# Dosimetric analysis of SIB whole brain radiotherapy planning: Comparison of coplanar VMAT and tomotherapy techniques

**DOI:** 10.1002/acm2.70313

**Published:** 2025-10-24

**Authors:** Eda Kaya Pepele, Öztun Temelli

**Affiliations:** ^1^ Yeşilyurt Vocational School Department of Electronics and Automation Biomedical Device Technology Program Malatya Turgut Özal University Malatya Turkey; ^2^ Department of Radiation Oncology Faculty of Medicine Inonu University Malatya Turkey

**Keywords:** brain metastases, CI, dosimetric analysis HI, PQI, tomotherapy, VMAT

## Abstract

**Background:**

Radiotherapy techniques have advanced significantly over the past few decades. Whole‐brain radiotherapy combined with a simultaneous integrated boost (WBRT+SIB) is increasingly used to treat limited brain metastases.

**Purpose:**

To retrospectively compare helical tomotherapy and coplanar volumetric modulated arc therapy (VMAT) for WBRT with a SIB‐WBRT in patients with multiple brain metastases. Additionally, it emphasizes the importance of selecting appropriate evaluation indices when comparing SIB plans.

**Materials and methods:**

Fifteen patients with 2‐ 3 metastatic lesions were retrospectively analyzed in this study. Treatment planning was performed using TomoHD and eclipse planning systems for tomotherapy and VMAT, respectively. Dose–volume histograms were used to assess the doses delivered to the target volumes and organs at risk (OARs). Quantitative metrics, including the homogeneity index (HI), conformity index (CI), and plan quality index (PQI), were used for the evaluation.

**Results:**

Tomotherapy yielded significantly higher D98% values for both the planning target volume (PTV) WB and PTV_met compared with VMAT (*p* < 0.05). It also provided lower D_max_ and D_mean_ values for the lenses and eyes (*p* < 0.001 and *p* < 0.02, respectively). Tomotherapy was superior in terms of PTV whole‐brain CI and PTV_met HI and CI (*p* < 0.05). However, no significant difference was observed in the PQI values between the techniques (*p* > 0.05).

**Conclusion:**

Both tomotherapy and VMAT achieved acceptable target volumes and OAR doses in SIB applications. Tomotherapy showed advantages in terms of dose conformity and critical organ sparing. Moreover, this study highlights the impact of selecting appropriate evaluation indices on interpreting plan quality, particularly for complex treatment approaches such as SIB.

## 1 INTRODUCTION

Treatment of brain metastases includes whole‐brain radiotherapy (WBRT), surgery, (SRS), and systemic therapy, and the patient's neurologic status, disease stage, and number, size, and location of metastases are among the key determinants of management.^1^

SRS, in addition to WBRT, improves lesion control and increases survival in patients with a single metastasis.^2^ Furthermore, for patients with 1–4 lesions, WBRT combined with SRS may improve intracranial lesion control.^3^ However, these applications may be limited by the number and size of metastases.^4^

Radiotherapy (RT) technology has recently evolved significantly in recent years. Compared with WBRT alone, intensity‐modulated radiotherapy (IMRT), image‐guided radiotherapy (IGRT), volumetric modulated arc therapy (VMAT), and helical tomotherapy (HT) systems, which are capable of helical IMRT, have significantly improved critical organ protection while achieving tumor‐appropriate dose distribution.^5^ Recently developed advanced radiation therapy techniques, such as HT, combine WBRT with a highly conformal boost dose to metastases during the treatment session, enabling dose‐enhanced treatment.^6^, ^7^ VMAT was developed based on IGRT technology. It features a new high‐precision accelerator, excellent inverse optimization treatment plan software, and precise dose verification equipment to achieve accurate positioning, planning, and treatment.^8^

With the advent of many new technologies, it is important to thoroughly understand the characteristics, advantages, and disadvantages of each technology and to choose an optimal plan to achieve the goal of precise RT. Recent comparisons of RT techniques for simultaneous integrated boost (SIB) treatments for multiple brain metastases in combination with WBRT have revealed several potential disadvantages and advantages. However, the quantities used to compare the treatment plans may be incomplete in terms of the volumes irradiated simultaneously at different doses. Recent retrospective series suggest that WBRT with a SIB achieves favorable intracranial control with acceptable toxicity. In a 107‐patient cohort, SIB‐WBRT yielded a median intracranial PFS of 13.4 months (6‐ and 12‐month iPFS 68.0% and 50.8%) and no observed radionecrosis; 6‐ and 12‐month local control rates were 84.3% and 73.3%, respectively.^9^ Comparative studies indicate better intracranial control with WBRT+SIB versus WBRT alone or WBRT+SRS in several settings, with grade ≥3 toxicity in the low single digits and generally similar toxicity profiles across arms.^10^ Meta‐ or review‐level summaries likewise report improved intracranial control with WBRT+SIB, though overall survival benefits are inconsistent, underscoring the need for prospective trials and integration with contemporary systemic therapies.^11^ This retrospective, single‐institution, within‐patient paired planning study compared HT and coplanar VMAT for SIB‐WBRT in patients with multiple brain metastases. Using the homogeneity and conformity (HI and CI) indices to define plan conformity, the healthy tissue CI in the case of multiple target volumes irradiated simultaneously, and the plan quality index (PQI) to evaluate target coverage and doses delivered to critical organs, we aimed to demonstrate some potential advantages and disadvantages between the techniques and contribute to the selection of the appropriate treatment modality.

## 2 MATERIALS AND METHODS

### 2.1 Patient selection

This was a retrospective, single‐institution, within‐patient paired planning study conducted in a radiation oncology department. We included 15 adult patients with multiple parenchymal brain metastases (2–3 lesions) for whom complete planning datasets were available. All cases had been treated previously in our department; the present analysis is dosimetric only (re‐planning on existing imaging without altering delivered care). For each patient, a planning CT (slice thickness ≤ 3 mm) was rigidly registered to contrast‐enhanced Magnetic Resonance Imaging (MRI), and identical contours were used to generate both HT and coplanar VMAT plans on the same dataset. Metastatic lesion volumes ranged from 5.7 to 33.1 cm^3^ (Table 1). Prespecified inclusion and exclusion criteria; eligible cases were adults (≥18 years) with 2–3 parenchymal brain metastases identified on contrast‐enhanced MRI. Inclusion required availability of a planning CT (slice thickness ≤3 mm) with adequate rigid CT–MRI registration and contours of planning target volume (PTV) whole brain (PTV_ WB) and boost PTV for metastases (PTV_met) in accordance with the institutional protocol, as well as historical planning data from our department suitable for dosimetric re‐planning. Patients were excluded if they had a single metastasis only or leptomeningeal disease; if imaging or contours were incomplete, CT–MRI registration was inadequate, or artifacts would compromise dosimetry; or if they were pediatric patients.

**TABLE 1:** Clinical characteristic of the patients.

Patient	Age	Gender	Mass region (localization)	Number of PTV_met_	PTV_met_ volume cc^3^ (total)
1	66	M	Left cerebellum, left temporal	2 met	14
2	51	M	Right Parietal, Right Vertex	2 met	31.9
3	62	M	Right Frontal, Left Cerebellum	3 met	13.5
4	41	F	Lateral cerebellar hemisphere, left high frontal lobe, right cerebellar hemisphere	3 met	26.2
5	55	M	Left middle temporal, Right occipital cortex	2 met	11.2
6	66	M	Right parietal, left parietal	2 met	13.8
7	72	M	Right cerebellar hemisphere, left cerebellar hemisphere	2 met	18.5
8	41	M	Right frontal, left parasagittal	2 met	17.9
9	36	F	Right temporal mass, left frontal, left occipital	3 met	10.1
10	80	M	Left frontal mass, right temporooccipital	2 met	15.8
11	71	M	Right temporal mass, left parietal mass	2 met	5.7
12	58	M	Left cerebellar, right paretoooccipital	2 met	19.3
13	67	M	Cerebellum lesion, left frontal lesion, left occipital lesion	3 met	15.8
14	47	F	Cerebellum lesion, left frontal lesion	2 met	33.1
15	72	F	Right temporal mass, left parietal mass	2 met	8.9

### 2.2 Statistical analyses

Statistical analyses were performed using IBM SPSS Statistics, version 25 (IBM Corp., Armonk, NY, USA). Normality was assessed with the Kolmogorov–Smirnov test together with inspection of skewness and kurtosis. Differences between two means in this within‐patient paired design were evaluated using two‐sided paired *t*‐tests, with *p* < 0.05 considered statistically significant. In addition to *p*‐values, we report effect sizes as Cohen's *d* for paired samples (d_z), consistent with the values presented in the tables.

### 2.3 Target volume and critical organ contouring

Computed tomography (CT) scans of the whole cranium with a slice thickness of 1 or 2 mm that had received prior RT were combined with the available contrast‐enhanced T1‐weighted MR images. The whole‐brain planned target volume (PTV_WB) and metastases (PTV_met) were contoured on the CT images. The target delineation criteria were the same for both groups: the GTV was defined as brain metastases, the clinical tumor volume was defined as whole brain tissue, and the PTV was defined as the whole brain by adding a 3 mm margin to the CTV. The total volume of PTV_met per patient ranged from 5.7 to 33.1 cm^3^ (Table 1). The organs at risk (OAR) included the brainstem, eyes, lenses, optic chiasm, and optic nerves.

### 2.4 Dose prescription and treatment planning

In the Tomotherapy Planning Workstation (TomoHDA, Accuray), a convolution/superposition dose‐calculation algorithm was used with a field width of 2.5 cm, pitch 0.287, and modulation factor 2.5 for a 6 MV photon beam. Dose calculation of VMAT plans was performed with an anisotropic analytical algorithm using a single isocentric two full arc (CW and CCW) technique at a dose rate of 600 MU/min at 6 MV photon energy on the Eclipse treatment planning system. Optimization to cover 95% of the PTV with 100% of the defined dose was considered as plan acceptance. The maximum (D_max_) and mean (D_mean_) doses of the oars were determined according to the dose constraints, and the maximum and minimum doses of the OARs were evaluated in the optic chiasm, left eye (L‐eye), right eye (R‐eye), left lens (L‐lens), right lens (R‐lens), left optic nerve (L‐optic nerve), right optic nerve (R‐optic nerve), and brainstem. For the treatment planning criteria of all patients included in the study, we used the study by Grosu et al. as a reference.^12^ The PTV criteria for the tomotherapy and VMAT treatment plans for the parameters of the reference study are presented in Tables 2 and 3, respectively. The dose restrictions for the OARs are presented in Table 4.

**TABLE 2:** Treatment plans (target volume planned for whole‐brain PTV_wholebrain_), target volume planned for metastasis PTV_met_).^9^

Radiotherapy planning	PTV_whole brain_	PTV_met_
Prescribed dose/fraction (Gy)	30 Gy/12frk	42 Gy/12frk

**TABLE 3:** Dose coverage criteria for planning target volumes (PTV_wholebrain_, PTV_met_).^9^

Parameters	According to the protocol	Small deviation	Large deviation
PTV whole brain (30Gy)	D_98%_ ≥ 25Gy	24Gy ≤ D_98%_ < 25Gy	D_98%_ < 24Gy
	D_mean_ ≤ 35 Gy	35Gy < D_mean_ ≤ 37 Gy	D_mean_ > 37 Gy
PTV_met_ (42Gy)	D_98%_ ≥ 39.9Gy	39.06 Gy ≤ D_98%_ < 39.9Gy	D_98%_ < 39.06

**TABLE 4:** Organs‐at‐risk (OAR) dose constraints.^9^

Parameters	According to the protocol	Small deviation	Large deviation
Optic nerve chiazma	D2% ≤ 33Gy	33Gy < D2% ≤ 35Gy	D2% > 35Gy
Lenses	D2% ≤ 7Gy	7Gy < D2% ≤ 10Gy	D2% > 10Gy
Eyes	D2% ≤ 33Gy	33Gy < D2% ≤ 35Gy	D2% > 35Gy
Brain stem	D2% ≤ 33Gy	33Gy < D2% ≤ 35Gy	D2% > 35Gy

D_98%_ (dose received by 98% of the PTV volume) _and_ D_2%_ (dose received by 2% of the PTV volume) were defined as the minimum and maximum doses, respectively. The data from the DVHs obtained from all the plans were analyzed.

#### 2.4.1 Optimization objectives and prioritization

HT and coplanar VMAT plans were performed on the same dataset by a single experienced planner according to a predetermined optimization hierarchy to ensure consistency. The predetermined optimization hierarchy was as follows: (1) adherence to absolute OAR constraints (e.g., serial organs D_max_) without exception; (2) prioritization of protocol target coverage for elevated boost PTV (PTV_met; target D_95_ ≥ 95% of prescription) and maintenance of PTV_WB coverage within tolerance (e.g., D_95_ ≥ 95%); (3) reduction of hot spots and improvement of homogeneity (D_2%_, HI); (4) improvement of CI using ring/normal tissue objectives. Once the targets were met, the homogeneity/conformity targets were relaxed within tolerance before any reduction in target coverage occurred. If an absolute OAR limit would otherwise be exceeded, minimal coverage relaxation was permitted and applied symmetrically to both techniques for the same patient; final acceptance was documented before dosimetric extraction. According to protocol, VMAT plans were coplanar (couch 0°); tomotherapy plans used ring structures (and directional blocking when necessary) to shape the dose falloff. Plan acceptability and clinical appropriateness were reviewed by a single radiation oncologist. Quantitative assessment used DVH‐derived endpoints (HI, CI, PQI, and OAR summaries) directly imported from the treatment planning system.

**FIGURE 1:**
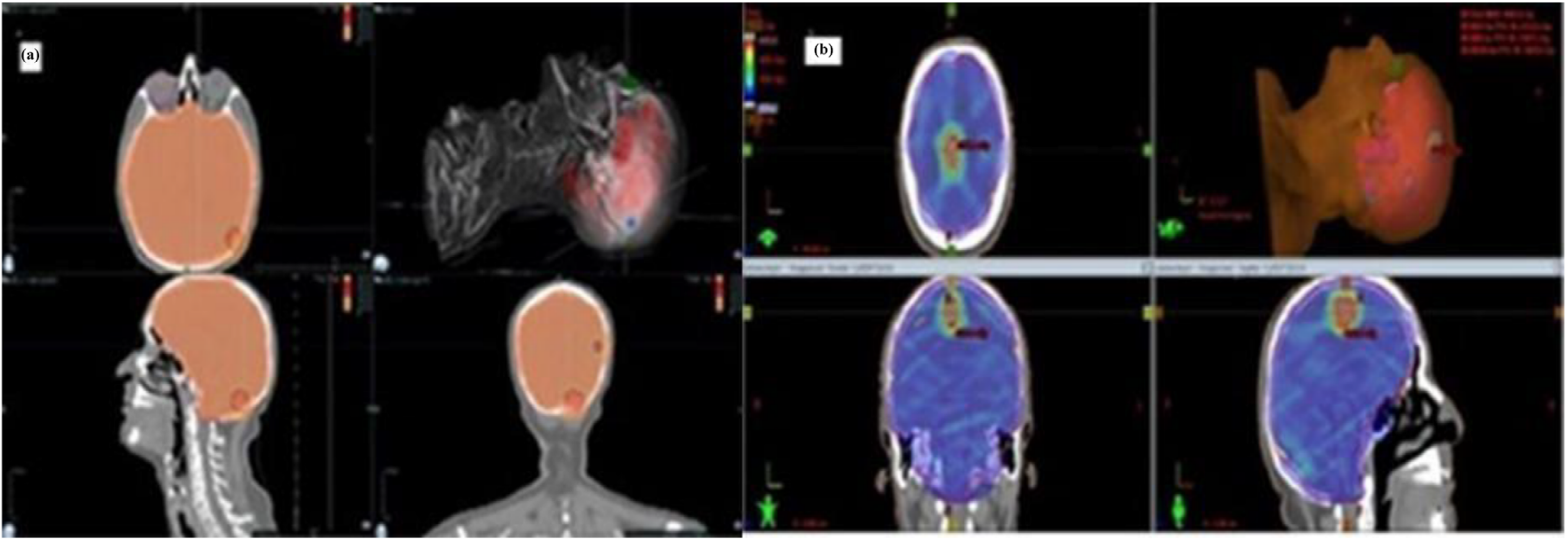
Representative axial dose distribution images for SIB planning in a patient with multiple brain metastases: (a) Helical Tomotherapy; (b) VMAT.

Figure 1 shows the dose distributions for the Tomo and VMAT SIB plans for a representative case with multiple brain metastases.

### 2.5 Plan comparison criteria

Dose–volume histograms (DVHs) were used to compare the target volume and critical organ doses (OARs) of treatment plans. For the target volumes (PTV___WB and PTV_met), D_98%_ and D_2%_ were considered the minimum and maximum doses, respectively. Treatment plans were optimized to minimize the dose to the OARs, while maximum dose coverage was desired for the PTV. Dose distribution characteristics were analyzed using the following quantitative parameters to measure target homogeneity and dose conformity.

#### 2.5.1 Homogeneity index (HI)

HI = (D_2_–D_98_)/D_median_, where D_median_ = D_50_, indicates the median target dose. HI = 0 indicates perfect target dose homogeneity.^13^

#### 2.5.2 Conformity index (CI)

CI = V_RI_/V_T_, defined as the ratio between the total volume V_RI_ and V_T_ surrounded by the reference isodose RI. A CI value close to one corresponds to a high degree of conformity.^14^

In addition to the HI and CI, which improve the ability to distinguish between the optimal plan and the quality of the plan, the PQI formula of Leugh et al. was used to better evaluate the agreement between the irradiated target volume and the volumes receiving the prescribed dose in the case of multiple simultaneously irradiated target volumes.

#### 2.5.3 Plan quality index (PQI)

There are three main indices: H, M, and P. The first is the healthy tissue index, H, which defines healthy tissue compliance and is a measure of the volume of healthy tissue included in the reference isodose. Second, M is a function used to define the target coverage and monitor the performance of hot and cold spots within the target area. Finally, *p* is the merit, expressed as the identification of a critical organ of interest. The mathematical expressions corresponding to the H, M, and P plan quality indices are presented below, respectively. Equation (4) shows the final index (i.e., the PQI), which represents the quality of a treatment plan and can be used to compare competing plans.^14^

(1) $$H=\frac{1}{r}x\sum_{i=1}^{r}\frac{\mathrm{TVRI},i}{VRI,i}$$

where

*r* is the number of target volumes assessed
TV_RI,i,_is the target volume covered by the ith reference dose
V_RI,i_ is the total isodose volume of the ith reference dose
(2) $$\begin{array}{c} M=\frac{1}{r}\sum_{j=1}^{r}\left\{\frac{\sum_{i=1}^{p}\left(\frac{V_{Tj_{1}Di}}{V_{Tj_{1}RDi}}\right)+\sum_{i=1}^{q}\left(1-\frac{V_{Tj_{1}Di}}{V_{Tj_{1}ADi}}\right)}{\sum_{i=1}^{p}\left(\frac{100}{V_{Tj_{1}RDi}}\right)+q}\right\} \end{array}$$

where

*r* is the number of targets with different prescription doses,
*p* is the number of cold spot checks,
*q* is the number of hot spot checks,
V_Tj,Di_ represents the volume of the jth target (in %) receiving a dose of at least the ith dose level,
V_Tj,RDi_ represents the minimum volume of the jth target (in %) receiving at least the ith dose level,
V_Tj,AD_ represents the allowable volume of the jth target (in %) receiving at least the ith dose level.
(3) $$P=\frac{1}{n}+\sum_{j=1}^{n}\left(\frac{1}{m}x\sum_{i=1}^{m}1-\frac{Voj,Di}{LVoj,ADi}\right)$$

where

*n* is the number of critical organs to be monitored,
*m* is the number of dose check points used for the jth critical organ,
V_Oj,Di_ represents the volume (in %) of the *j*th critical organ receiving at least the *i*th dose level,
V_Oj,ADi_ represents the allowable volume (in %) of that organ receiving at least the *i*th dose level.
(4) $$\mathrm{PQI}=\sqrt{\left({\left(1-H\right)}^{2}+{\left(1-M\right)}^{2}+{\left(1-P\right)}^{2}\right)}\;$$

In the worst case, H = 1, M = 1, and P = 1 (i.e., [1, 1, 1]), yielding PQI = √3; this value can be interpreted as the plan's distance from the ideal with respect to these parameters.^14^ In our study, while calculating the PQI, hot and cold points were defined for M merit. For the cold control point, at least 95% of the target volume with a minimum PTV dose requirement received 100% of the prescribed dose, and for the hot control point, volumes receiving 107% of the defined dose were considered. In the analysis of the critical organ merit (*p*) value, the brain stem, lenses, optic nerves, and optic chiasm were selected as critical organs, and the dose limits of critical organs in the study by Grosue et al. were used as a reference.^12^

## 3 RESULTS

### 3.1 Quantitative evaluation of target volumes

Table 5 shows the standard deviations and statistical comparison results of the target volume criteria of D_98%_ and D_2%_ for the SIB planning technique using the tomotherapy and VMAT techniques with PTV_WB 30 Gy/12 frk to the whole brain and PTV‐met 42 Gy/12 frk to the metastases.

**TABLE 5:** Statistical analysis results of PTV criteria.

Parameters	Mean	St. deviation	*t*	*p*
Tomo D_%98_ **(30Gy)**	30.36	1.93	2.339	**0.035**
VMAT D_%98_ **(30Gy)**	29.12	0.35		
Tomo D_%2_ **(30Gy)**	34.94	2.56	−2.154	**0.049**
VMAT D_%2_ **(30Gy)**	36.58	2.29		
Tomo D_%98_ **(42Gy)**	41.65	0.42	4.586	**<0.001**
VMAT D_%98_ **(42Gy)**	40.33	0.81		
Tomo D_%2_ **(42Gy)**	43.22	0.31	0.320	0.754
VMAT D_%2_ **(42Gy)**	43.14	0.71		

*Note*: D_98%_ = doses received by 98% of the PTV, representing the minimum dose to the PTV; D _2%_ = doses received by 2% of the PTV. Statistical significance was set at ***p* < 0.05**.

**FIGURE 2:**
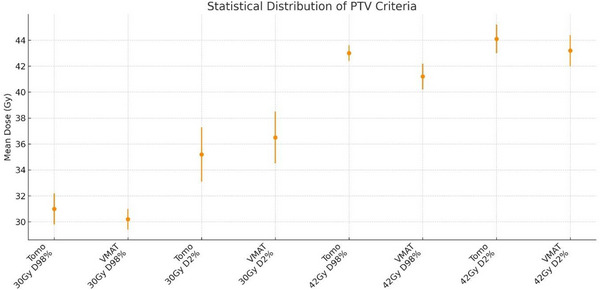
PTV dosimetric endpoints for SIB‐WBRT. Points show the mean across patients (*n* = 15); error bars denote the standard deviation (SD). Endpoints are D98% (near‐minimum) and D2% (near‐maximum) for PTV_WB (30 Gy) and PTV_met (42 Gy) in tomotherapy versus VMAT. Doses in Gy; paired *t*‐tests, significance threshold *p* < 0.05.

According to Table 5, for PTV_WB (30 Gy), tomotherapy achieved higher D98% (30.36 ± 1.93 Gy vs. 29.12 ± 0.35 Gy; *p* = 0.035) and lower D2% (34.94 ± 2.56 Gy vs. 36.58 ± 2.29 Gy; *p* = 0.049) than VMAT. For PTV_met (42 Gy), D98% was significantly higher with tomotherapy (41.65 ± 0.42 Gy vs. 40.33 ± 0.81 Gy; *p* < 0.001), whereas D2% did not differ between techniques (43.22 ± 0.31 Gy vs. 43.14 ± 0.71 Gy; *p* = 0.754). These differences are visualized in Figure 2 (points = mean, error bars = SD, *n* = 15; doses in Gy).

### 3.2 OARs

The results of the statistical analysis of the maximum and mean dose values of the tomotherapy and VMAT treatment plans for the critical organs are presented in Tables 6 and 7.

**TABLE 6:** Statistical analysis results of the maximum dose (D_max_) values for the critical organs.

OAR	Mean	St. deviation	*t*	*p*
Tomo right lens	6.24	0.52	−9.775	**<0.001**
VMAT right lens	9.03	0.79		
Tomo left lens	6.06	0.48	−10.864	**<0.001**
VMAT left lens	8.68	0.83		
Tomo right eye	24.08	2.13	−5.145	**<0.001**
VMAT right eye	28.47	1.94		
Tomo left eye	24.26	1.81	−2.473	**0.027**
VMAT left eye	26.10	3.13		
Tomo right optic nerve	29.54	1.17	−2.078	0.057
VMAT right optic nerve	30.51	0.85		
Tomo left optic nerve	29.28	1.33	−3.781	**0.002**
VMAT left optic nerve	30.59	1.05		
Tomo chiasma	31.66	2.33	−1.221	0.242
VMAT chiasma	32.18	1.44		
Tomo brainstem	32.96	2.42	0.062	0.951
VMAT brainstem	32.93	2.30		

*Note*: Statistical significance was set at ***p* < 0.05**.

**TABLE 7:** Statistical analysis of the D_mean_ values of the critical organs.

OAR	Mean	SD	*t*	*p*
Tomo right lens	5.12	0.53	−12.531	**<0.001**
VMAT right lens	8.16	0.79		
Tomo left lens	5.03	0.58	−11.383	**<0.001**
VMAT left lens	7.69	0.70		
Tomo right eye	12.32	1.29	−3.303	0.004
VMAT right eye	14.81	2.67		
Tomo left eye	12.60	0.96	−3.740	**0.002**
VMAT left eye	14.79	2.44		
Tomo right optic nerve	25.58	2.68	0.535	0.601
VMAT right optic nerve	25.08	1.97		
Tomo left optic nerve	25.71	2.85	1.500	0.156
VMAT left optic nerve	24.71	2.54		
Tomo chiasma	29.99	2.98	.281	0.783
VMAT chiasma	29.70	1.43		
Tomo brainstem	31.47	1.20	2.089	0.055
VMAT brainstem	30.88	1.11		

*Note*: Statistical significance was set at ***p* < 0.05**.

**FIGURE 3:**
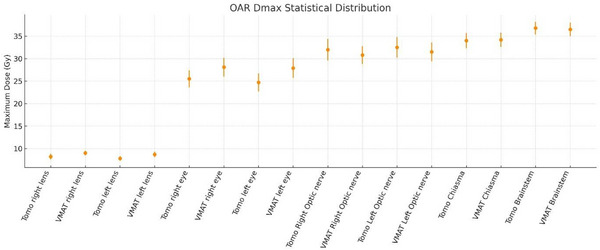
OAR maximum dose (D_max_) for tomotherapy versus VMAT. Points show the mean across patients (*n* = 15); error bars denote the standard deviation (SD). Doses in Gy; paired *t*‐tests, significance threshold *p* < 0.05. Tomotherapy is plotted with circles, VMAT with squares.

In the D_max_ evaluation of OARs in both planning techniques using the tomotherapy and VMAT techniques, when the maximum dose values of the lenses, eyes, and left optic nerve were compared, the mean values of tomotherapy were significantly lower than the mean values of the VMAT plans, indicating that the maximum dose to the lenses was significantly lower in the Tomotherapy plans (*p* < 0.001, *p* < 0.001, *p* < 0.01, *p* < 0.02). No statistically significant difference was found in the maximum dose to the right optic nerve, brainstem, or optic chiasm between the two techniques (*p* = 0.057, *p* = 0.242, and *p* = 0.951, respectively). These findings are depicted in Figure 3 (means with SD error bars; *n* = 15; doses in Gy).

Table 7 shows the results of the statistical analysis of the mean dose values of the tomotherapy and VMAT treatment plans for the critical organs.

**FIGURE 4:**
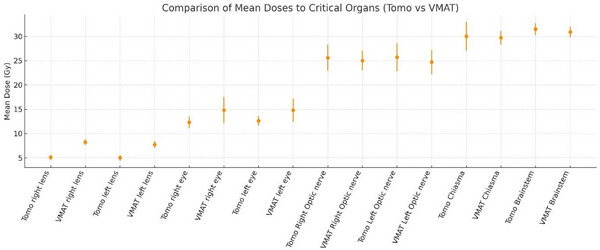
OAR mean dose (D_mean_) for tomotherapy versus VMAT. Points show the mean across patients (*n* = 15); error bars denote the standard deviation (SD). Doses in Gy; paired *t*‐tests, significance threshold *p* < 0.05.

According to Table 7, Tomotherapy yielded significantly lower mean doses (D_mean_) to the lenses (bilateral; both *p* < 0.001) and the eyes (right *p* = 0.004; left *p* = 0.002) compared with VMAT. No significant differences were observed for the optic nerves (right *p* = 0.601; left *p* = 0.156), optic chiasm (*p* = 0.783), or brainstem (*p* = 0.055). These findings are depicted in Figure 4 (means with SD error bars; *n* = 15; doses in Gy).

### 3.3 Plan evaluation criteria

The H, M, P, and PQI values ​​for the Tomotherapy and VMAT treatment plans are listed in Table 8. The statistical analysis results for 30 and 42 Gy (HI, CI, and PQI) are presented in Table 9.

**TABLE 8:** *H*, *M*, *P*, and PQI values for the Tomo and VMAT plans.

Patient	*H* (Tomo)	*H* (VMAT)	*M* (Tomo)	*M* (VMAT)	*p* (Tomo)	*p* (VMAT)	Tomo (PQI)	VMAT (PQI)
**1**	0.6891	0.4500	1.0000	0.9890	0.9949	0.9947	0.311	0.550
**2**	0.7086	0.8400	1.0000	1.0000	0.9937	0.9106	0.291	0.168
**3**	0.6539	0.7567	1.0000	1.0000	0.9880	0.9935	0.346	0.243
**4**	0.7182	0.5750	1.0000	1.0000	0.9950	0.9953	0.281	0.425
**5**	0.7032	0.9707	1.0000	0.9990	0.9945	0.9947	0.296	0.388
**6**	0.7210	0.9451	1.0000	1.0000	0.9950	0.9890	0.279	0.412
**7**	0.8680	0.8818	1.0000	1.0000	0.9890	0.9940	0.132	0.118
**8**	0.8220	0.8519	1.0000	1.0000	0.9885	0.9822	0.178	0.385
**9**	0.8627	0.6842	1.0000	1.0000	0.9990	0.9700	0.137	0.562
**10**	0.5864	0.6842	1.0000	1.0000	0.9930	0.9765	0.413	0.348
**11**	0.7886	0.8891	1.0000	1.0000	0.9230	0.9170	0.221	0.118
**12**	0.9400	0.9001	1.0000	1.0000	0.9950	0.9820	0.198	0.100
**13**	0.7839	0.7598	1.0000	1.0000	0.9885	0.9850	0.216	0.240
**14**	0.7526	0.8659	1.0000	1.0000	0.9850	0.9821	0.248	0.134
**15**	0.7881	0.8588	1.0000	1.0000	0.9858	0.9839	0.212	0.141

**TABLE 9:** Statistical analysis results for the planning criteria.

PTV evaluation criteria	Mean	SD	*t*	*p*
Tomo HI (30 Gy)	0.30	0.08	−0.132	0.897
VMAT HI (30 Gy)	0.31	0.10		
Tomo CI (30 Gy)	0.98	0.00	3.650	**0.003**
VMAT CI (30 Gy)	093	0.05		
Tomo HI (42 Gy)	0.03	0.01	−7.119	**<0.001**
VMAT HI (42 Gy)	0.07	0.02		
Tomo CI (42 Gy)	0.99	0.03	3.395	**0.004**
VMAT CI (42 Gy)	0.92	0.07		
Tomo PQI	025	0.08	−0.904	0.381
VMAT PQI	0.29	0.16		

*Note*: ***p* < 0.05** was considered statistically significant.

As shown in Table 8, both treatment planning techniques are qualified plans, and the PQI results meet all the requirements determined by the radiation oncologist for both techniques. The (H, M, P) values were found to have acceptable plan criteria for both planning techniques away from the (1,1,1) scenario. The results of the comparison indices of the treatment plans are listed in Table 9.

**FIGURE 5:**
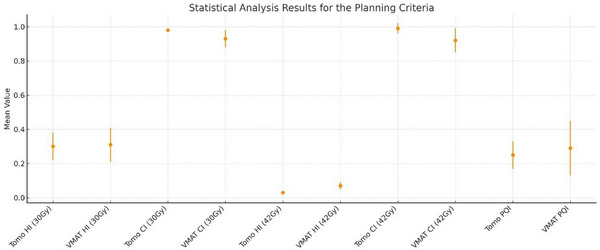
Plan metrics per Table 9—HI and CI at 30 Gy (PTV_WB) and 42 Gy (PTV_met), and PQI for Tomotherapy versus VMAT. Points show the mean across patients (*n* = 15); error bars denote the standard deviation (SD). Metrics are unitless. Tomotherapy is plotted with circles and VMAT with squares.

As shown in Table 9, while no statistically significant change was observed in the HI values of the Tomotherapy and VMAT treatment plans in the 30 Gy treatment planning given to the whole brain, the Tomotherapy plans had significantly greater CI values than the average values of the VMAT plans and were closer to the ideal plan criteria (*p* < 0.05). The HI and CI values of the 42 Gy tomotherapy treatment plans for metastases were significantly closer to the ideal plan criteria than those of the VMAT plans (*p* < 0.05). Despite significant differences in some component indices (HI/CI at 42Gy), the composite PQI was comparable between techniques (tomotherapy 0.25 ± 0.08 vs. VMAT 0.29 ± 0.16; paired *t* = −0.904, *p* = 0.381; unitless), supporting overall plan acceptability for both modalities. See Figure 5.

## 4 DISCUSSION

In patients with multiple metastases, the SIB technique for dose escalation reduces the number of treatment sessions.^15^ Recently developed advanced radiation therapy techniques, such as HT, combine WBRT with a highly conformal boost dose to metastases in a single treatment session, enabling dose‐escalated treatment compared with WBRT alone.^16^ Moreover, the VMAT approach, which is an advanced radiation technique, is effective in the planning and treatment of multiple metastases while achieving clinically acceptable dosimetry in terms of dose conformity and a low‐dose spillage.^17^ Taken together, contemporary series indicate that SIB‐WBRT can provide durable intracranial control with low rates of high‐grade toxicity, underscoring the clinical relevance of optimizing plan conformity and homogeneity in the SIB setting.^12^, ^13^, ^14^ In this study, both tomotherapy and VMAT techniques complied with the planning criteria specified for the SIB technique in the planning of multiple brain metastases. When the results obtained from the treatment plans were compared, better D_98%_ dose coverage of the whole‐brain target volume (PTV whole brain) and the lowest D_2%_ dose values were obtained with tomotherapy Table 3 (*p* < 0.05). In SIB plans with 42 Gy for metastases (PTV_met), tomotherapy was superior in terms of target volume coverage at D_98%_, whereas there was no statistically significant difference between the planning techniques in the maximum dose evaluation at D_2%_ (*p* > 0.05). Sun et al. compared whole‐brain and SIB treatment plans in patients with Grade II gliomas using HT, VMAT, and IMRT techniques and reported that tomotherapy plans were significantly better than VMAT and IMRT in terms of D_98%_, with D_2%_ values in line with our results.^8^

When the D_max_ and D_mean_ of the critical organs were evaluated, the maximum doses of tomotherapy for the lenses and eyes were significantly lower than those of the VMAT plans (Table 4; *p* < 0.05). The difference observed in the eyes and lenses compared with the VMAT plans may be attributed to the rapid decrease in tomotherapy dose. Similarly, Sun et al. reported that lens doses had lower mean and maximum doses with tomotherapy than with VMAT.^8^ Hauswald et al. concluded that the special design of the device can achieve comparable or better plan quality than conventional methods used for a limited number of brain metastases via tomotherapy.^18^ In our study, the superiority of tomotherapy allowed optimization in terms of reducing critical organ doses because of its ability to cover the target volume.

Among the quantitative methods used to compare tomotherapy and VMAT plans, HI and CI are indicators of the homogeneity of the target volume, whereas CI focuses on the relationship between the shape of the reference isodose and PTV. PQIs are obtained by weighing the DVH values and are based on a comparison of DVH‐derived measurements using patient group‐specific protocols.^14^, ^15^, ^16^, ^17^, ^18^, ^19^ In this study, when target volumes, critical organs, and plan‐specific dosimetric parameters were considered in the PQI evaluation and the arithmetic of all components was considered for simultaneous evaluation, no statistically significant difference was found between the two RT techniques (*p* > 0.05). This may be attributed to the fact that the planning criteria determined for the H, M, and P merits in the PQI comparison yielded similar results in both plans. For example, when defining M merit, 95% of the PTV receiving 95% of the prescribed dose was determined to be the minimum requirement for target coverage. Accordingly, when optimizing the treatment plans in our study, optimization to cover 95% of the PTV with 100% of the prescribed dose was an important criterion for plan acceptance, and both techniques satisfied this criterion. However, when the CI results of the PTV_WB and the HI and CI values of the PTV_met were compared, the tomotherapy plans were superior, and a statistically significant difference was found between the two techniques (*p* < 0.05). Although studies have shown the superiority of tomotherapy in terms of dose homogeneity and conformity, Sun et al. reported no statistically significant difference between the techniques in terms of homogeneity and conformity in the comparison of VMAT, Tomotherapy, and IMRT treatment plans in patients with brain metastases.^8^ This difference in the CI index in our study may be due to the high conformity of tomotherapy, as well as the fact that the comparison metrics (HI and CI) are affected by the dose and volume parameters due to the lack of satisfactory results when the targeted dose and related volume concepts are not well defined in SIB treatment techniques. This is because when there are different prescription doses and two targets in a plan, the isodose line of two different dose levels may not have the same volume as the targets. Most index definitions in plan evaluation provide satisfactory CI values for higher‐dose targets but fail to meet other targets in SIB treatment plans.^20^ Helical delivery of tomotherapy provides denser angular sampling and higher modulation than coplanar VMAT, resulting in more uniform dose painting around multiple off‐axis targets under whole‐brain coverage. Narrow longitudinal jaws and dynamic jaw motion tighten the cranio‐caudal penumbra and smooth boost‐WBRT couplings, reducing D_2_% and improving HI. In contrast, coplanar VMAT must balance arc geometry, collimator angle, and leaflet motion/tongue‐and‐groove constraints; with multiple spatially separated foci, these limitations may necessitate modest compromises in homogeneity or yield slightly larger high‐dose isodose volumes. These mechanisms likely explain the superior HI/CI we observed with tomotherapy and are consistent with previous cranial dosimetric reports supporting helical delivery for dose uniformity and conformability.^8^, ^18^

Non‐coplanar VMAT as a potential improvement. Beyond the coplanar VMAT and HT plans analyzed here, non‐coplanar VMAT (via couch‐kick partial arcs or automated platforms such as HyperArc) has been shown to sharpen dose fall‐off and improve conformity relative to coplanar VMAT in cranial SRS, e.g., lower gradient index and higher CI with ncVMAT than cVMAT.^21^ Automated HyperArc implementations further report reduced brain V5/V12/V24 and improved conformity versus coplanar RapidArc in cranial FSRT contexts.^22^ These potential gains, however, come with added delivery complexity: couch rotations may add setup steps/time and require collision assessment/avoidance during planning and delivery.^23^ As non‐coplanar plans were not evaluated here, head‐to‐head comparisons with tomotherapy and coplanar VMAT are warranted in future SIB‐WBRT studies. Delivery efficiency and resource utilization. Contemporary reports consistently show shorter delivery times with coplanar VMAT than with HT (e.g., median beam‐on ≈6–7 min vs. ≈12–16 min in comparable protocols), reflecting the efficiency of a few high‐output arcs; TomoEDGE dynamic jaws can shorten HT delivery but typically do not eliminate the time gap.^24^, ^25^ Beyond time, VMAT is widely implemented on conventional C‐arm linacs with established QA workflows, whereas tomotherapy is a dedicated platform with modality‐specific QA; overall throughput and total MUs remain platform‐dependent.^26^

This study is limited by its retrospective, single‐institution design and modest sample size (*N* = 15). Although a within‐patient paired planning approach was used to mitigate case‐mix variability between techniques, the cohort size constrains power to detect small between‐technique differences; accordingly, we report standardized effect sizes with 95% confidence intervals and interpret non‐significant results cautiously. To enhance consistency, all plans were generated by a single planner and reviewed by a single attending radiation oncologist; while this approach reduces inter‐observer variability, formal blinding and multi‐planner reproducibility testing were beyond the scope of this study. Another limitation is the study's exclusive focus on dosimetric parameters; future work should incorporate machine‐specific factors (treatment time, low‐dose exposure volumes, total MUs, QA pass rates) for a more comprehensive assessment. Finally, non‐coplanar VMAT plans were not evaluated here and should be examined prospectively alongside tomotherapy and coplanar VMAT to clarify dosimetric trade‐offs and clinical feasibility.

In conclusion, both techniques achieved clinically acceptable target coverage and OAR sparing for SIB‐WBRT in multiple brain metastases. Tomotherapy demonstrated superior dose homogeneity and conformity for both whole‐brain and metastatic targets, suggesting a potential advantage for dose‐escalated strategies while minimizing exposure to critical structures. The choice of evaluation metrics substantially influences comparative outcomes; integrating comprehensive indices such as PQI alongside classical measures (e.g., HI, CI) provides a more objective and holistic appraisal of plan quality.

## AUTHOR CONTRIBUTIONS

Eda Kaya Pepele: Generating ideas and hypotheses for the article, planning method, treatment planning and data organization and calculation, statistical analysis, reference source scan, writing, and editing. Öztun Temelli: Drawing patient contours, treatment planning evaluation, interpreting data, and contributing to the writing of the main sections of the article.

## CONFLICT OF INTEREST STATEMENT

The authors declare no conflicts of interest.

## ETHICS STATEMENT

This retrospective, single‐institution, within‐patient paired planning study was performed in one academic radiation oncology department under institutional ethics approval (decision no. 2024/4495).

## Data Availability

De‐identified planning data and analysis outputs are available from the corresponding author upon reasonable request.
